# Transverse Asymmetries of the Maxilla Even in Healthy and Apparently Symmetrical Subjects

**DOI:** 10.3390/ijerph18020446

**Published:** 2021-01-08

**Authors:** Giuseppe Currò, Giuseppa Bilello, Pietro Messina, Giuseppe Alessandro Scardina

**Affiliations:** Department of Surgical, Oncological and Stomatological Disciplines (DiChirOnS), University of Palermo, 90133 Palermo, Italy; giuseppe.curro@unipa.it (G.C.); giuseppa.bilello@unipa.it (G.B.); pietro.messina01@unipa.it (P.M.)

**Keywords:** transverse asymmetries, maxilla

## Abstract

In the formulation of an orthodontic treatment plan, the three-dimensional analysis of the dental arches represents a fundamental moment for the evaluation of all the morphological parameters necessary in order to have a correct and complete diagnosis. In this regard, the study of the dental arches on the horizontal plane is sometimes neglected or not thorough enough. When evaluating the transverse dimensions of the dental upper arche, the presence of an asymmetry is frequently found, and it means that an hemiarch is larger than the other. Furthermore, any variation in one of the three planes of space always involves an alteration also on the other two planes in order to have compensation. The morphology of a bone segment depends on various factors, mainly genetical, acquired and environmental. Regarding the environmental factors, the function determines the morphology, but this in turn determines the function. In the case of unilateral mastication, the upper maxilla will be asymmetrical, so growing patients will have compensation on other skull bones. From these considerations arises the need for a careful study of the horizontal plane of the maxilla in the presence of a malocclusion during diagnostic evaluations. These asymmetries, however, must be evaluated and quantified in the diagnostic phase in order to formulate a correct plan of treatment. The aim of this work is to demonstrate that there are almost always transverse asymmetries of the maxilla, albeit of a slight entity, even in healthy and apparently symmetrical subjects.

## 1. Introduction

The human body at birth undergoes development with bilateral symmetry. So, the right and left sides can be divided into identical mirror images. However, when the functions start due to biological factors inherent to processes of development as well as environmental disturbances, perfect bilateral symmetry is rarely found. Regarding the skull bones and the face, the symmetry of the craniofacial complex and its variation is due to several factors. Thiesen et al. [1] proposed a current review and they stated that in case of asymmetry, the etiology should be carefully investigated in order to achieve an adequate treatment plan. Chou [2] found that the normal pediatric face is asymmetric and the panel assessment of facial fluctuating asymmetry was influenced by the observers. This work suggested that a facial asymmetry needs careful investigation of the underlying etiology and comprehensive clinical examination in conjunction with imaging studies for etiopathogenesis, diagnosis, and localization of asymmetry. All individuals have a certain degree of asymmetry. Facial asymmetry is common in the overall population and is often presented subclinically. Cao [3] proposes a new facial asymmetry index in order to efficiently quantify the degree of facial asymmetry from 3D Computed Tomography. From a clinical point of view, the involvement of skeletal, dental, functional and soft tissue of craniofacial structures is strictly necessary. An assessment of structural and functional status of the patient by means of thorough clinical examination, comprehensive radiographic survey is indispensable for accurate diagnosis of asymmetries. Regarding this, Jackson [4] found that orthodontists showed expertise in assessing face symmetry compared with both laypersons and general dentists. Cheong [5] underline the importance of a complete diagnosis, because most

Patients notice horizontal or transverse discrepancy more often than vertical and sagittal asymmetry. So, clinical examination needs to be supplemented by other diagnostic modalities such as dental casts. Peck [6] found that cephalometric data obtained from the relative radiograms also show skeletal asymmetries in children and adults whose faces are considered aesthetically pleasing.

Azevedo [7] emphasized that dental arch asymmetry is present in most individuals from various populations, although those with malocclusion tend to have more asymmetry. Gateno et al. [8] produced a reliable estimation of fluctuating facial asymmetry in symmetrical subjects with a class I occlusion. They concluded that all individuals have some degree of asymmetry, so the resultant asymmetry is the result of stressors that could modify a genetic program. Any stressor can modify genes, epigenetic mechanisms, or the environment. Therefore, dental arches are surely modified by the function or by the environment of the skull. All these factors could lead to form changes and asymmetries, including of dental arches. Adaptations and adjustment are very frequent in the occlusion that very often is asymmetric.

For example, populations with a good occlusion in permanent dentition and any orthodontic treatment usually have about 1.0 mm of transverse or anteroposterior asymmetry of the dental arch, while it is more frequent in untreated subjects with Class II Division II malocclusion. In the opinion of Ghafari [9] and Singh [10], a slight nonpathologic asymmetry is usually invisible and often considered normal, but owing to the subjectivity of facial esthetics, the threshold of its clinical significance cannot be easily determined; so, the acceptability could depend on the region of asymmetry, clinician’s sense of balance and the patient’s perception of imbalance. First Van Valen [11], and then various authors such as Graham [12] and more recently Rolfe [13] and Cho [14], investigated on the so-called “fluctuating asymmetry”, as random variations in symmetry, within limits, have been recognized as normal.

Vig and Hewitt [15] have suggested that the dentoalveolar region is more adaptable and shows a greater degree of symmetry than the rest of the face, probably due to compensatory mechanisms which are able to compensate for the presence of unbalancing factors. However, if the occlusion does not respect the parameters of the determinants, a functional asymmetric activity could occur. This is an adaptation mechanism, that would lead to a deviation in the developmental age, or, if this compensation fails, a dysfunction in adulthood will occur.

Other authors clinically assessed dental asymmetry by comparing reference points on the occlusal surface of the corresponding plaster models.

In this regard, Thomas J. Maurice [16] carried out a study in order to describe the degree and distribution of the asymmetry of the dental arch. He considers the possible relationship between intra-arch and inter-arch asymmetry in a sample of Caucasian children with relatively intact mixed dentition. Škrinjarić et al. [17] assessed the fluctuating asymmetry of dental arch dimensions in orthodontic patients by scanning 3D virtual models. They found that measurements obtained on 3D models can be considered reliable and comparable to those obtained with digital calipers in conventional way. Both methods showed a high degree of concordance and reproducibility, as previously confirmed by Bootvong [18]. They concluded that the arch asymmetry was greater in the mandible than in the maxilla in all malocclusion groups.

Ferrario [19], and then also Alarcòn [20], investigated the electromyographic instrumental study of the masticatory muscles in patients with crossbite, and they found a great variability. They found that the altered occlusal relationship affected the coordination of the masticatory muscles when chewing on both sides. Furthermore, the functional alteration was more evident when the side with the altered morphology was directly involved, i.e., when chewing was performed on the crossbite side.

Therefore, a unilateral contraction of the maxilla is often the cause of posterior crossbite.

An example can be found in a study by Thilander and Lennartsson [21], who verified the possibility of identifying some specific occlusal and skeletal characteristics in patients who had deciduous dentition with posterior crossbite and had already been subjected to successful treatment, compared to those with early permanent dentition who had not undergone correction.

## 2. Materials and Methods

This study was performed on dental arches plaster models of orthodontic patients observed at the Department of Orthodontics of the “Paolo Giaccone” University Polyclinic in Palermo.

The study protocol conformed to the ethical guidelines of the 1964 Declaration of Helsinki and its later amendments or comparable ethical standards and was approved by the Institutional Review Board of the University of Palermo General Hospital (“Paolo Giaccone” University Polyclinic; approval number 11/2019).

The material consisted of patients all in a phase of developmental growth and there were 72 females and 72 males, aged between 11 and 13 years.

About the occlusal characteristics, all the patients showed a normocclusion: Angle’s Class I on the molars and canines, overjet about 2 mm and overbite about 30%, soft curve of Spee, no facial asymmetries and a good profile. All the subjects were healthy and apparently symmetrical.

### 2.1. Sample Selection

Patients were selected according to the following inclusion criteria:

- Absence of craniofacial malformations and/or associated syndromes;
- Absence of previous maxillofacial surgery;
- Absence of trauma;
- Absence of previous orthodontic treatment (mobile and/or fixed);
- The mastication was tested with a simpl gummy candy for 30 s, so the chewing side both by the orthodontist and the patient were noted.

The symmetry or eventual asymmetry of the two halves of the maxilla of each patient with respect to the median palatine raphe, both anteriorly and posteriorly, was measured and analyzed. For this purpose, the transverse diameter of the arch was measured. The measurements were obtained with:

- A fine-tipped digital caliper;
- A transparent millimeter grid.

After having traced the median palatine raphe on the plaster model, defined by a straight line passing through the meeting point of the second group of palatal folds with the raphe and a posterior transition point from the hard to the soft palate, the transparent millimeter grid was superimposed onto the model in order to compare the transverse diameters of the two hemiarches.

For the measurements of the transversity of the two hemi-maxillaries, a "Delta Orthodontics" digital caliper with fine tips was used and the following linear parameters were measured, by marking the second decimal place.

### 2.2. Inter-Premolar Diameter (mm)

**Figure 1:**
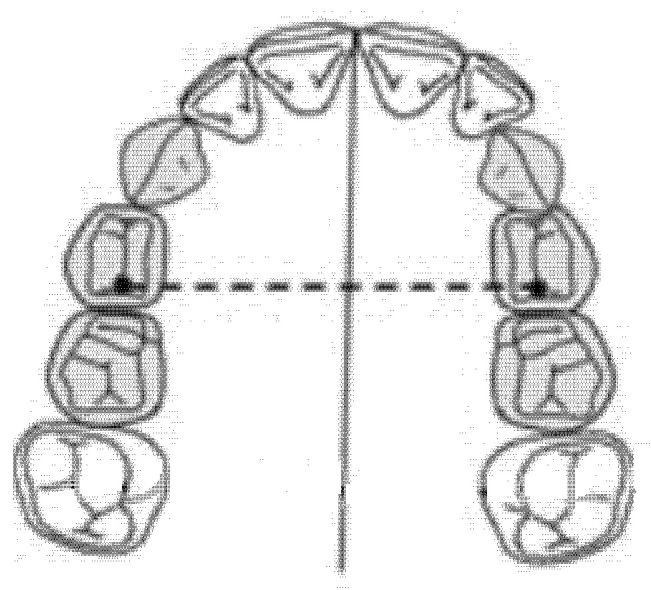
Inter-premolar diameter (mm).

The inter-premolar diameter was measured as a linear distance between the central fossae of the right first premolar and the left first premolar of the upper maxilla (Figure 1).

### 2.3. Intermolar Diameter (mm)

**Figure 2:**
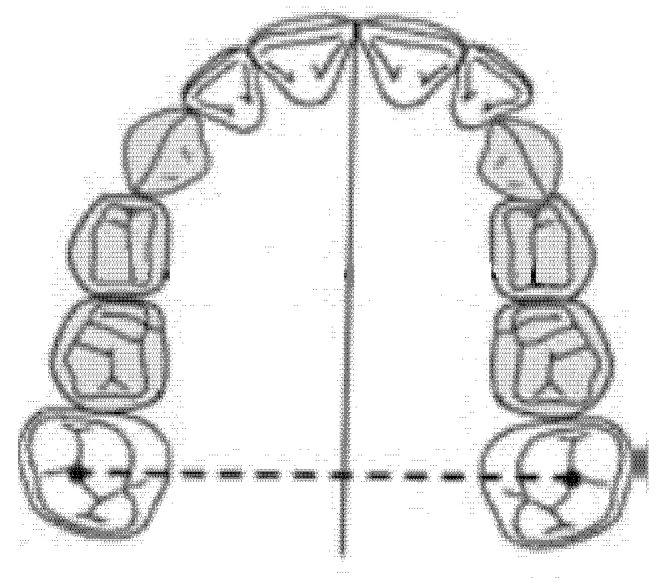
Intermolar diameter (mm).

The intermolar diameter was measured as a linear distance between the central fossae of the right first molar and the left first molar of the upper maxilla (Figure 2). 

### 2.4. Diameter of the Right Anterior Hemiarch (mm)

**Figure 3:**
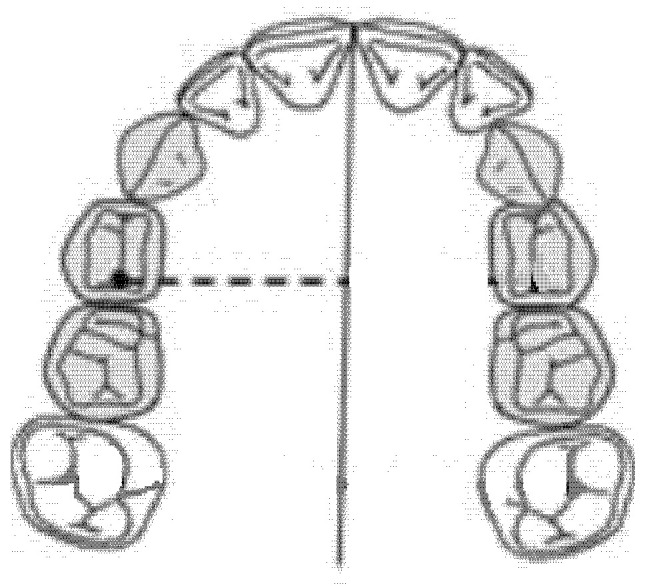
Diameter of the right anterior hemiarch (mm).

The diameter of the right anterior hemiarch was measured as a linear distance between the central fossa of the right first premolar and the median palatine raphe of the upper maxilla (Figure 3).

### 2.5. Diameter of the Left Anterior Hemiarch (mm)

**Figure 4:**
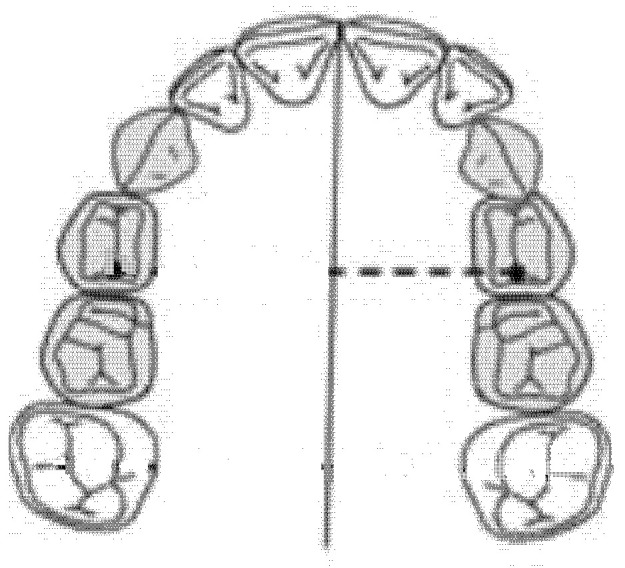
Diameter of the left anterior hemiarch (mm).

The diameter of the left anterior hemiarch was measured as a linear distance between the central fossa of the left first premolar and the median palatine raphe of the upper maxilla (Figure 4).

### 2.6. Diameter of the Right Posterior Hemiarch (mm)

**Figure 5:**
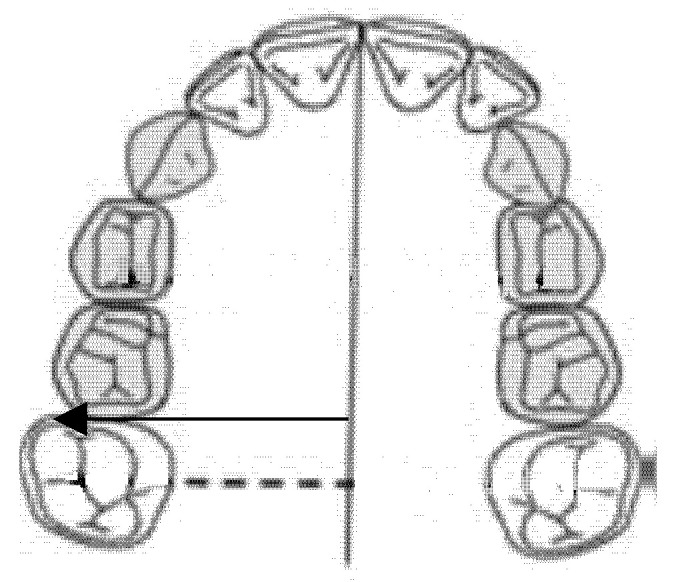
Diameter of the right posterior hemiarch (mm).

The diameter of the right posterior hemiarch was measured as a linear distance between the central fossa of the right first molar and the median palatine raphe of the upper maxilla (Figure 5).

### 2.7. Diameter of the left Posterior Hemiarch (mm)

**Figure 6:**
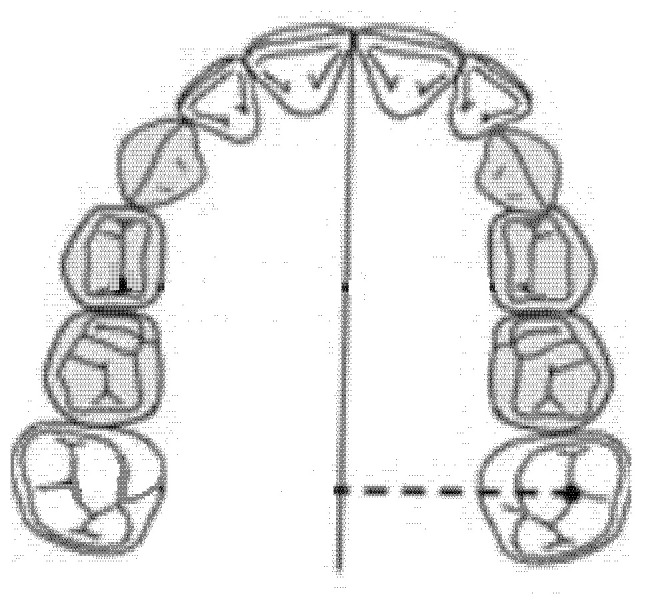
Diameter of the left posterior hemiarch (mm).

The diameter of the left posterior hemiarch was measured as a linear distance between the central fossa of the first left molar and the median palatine raphe of the upper maxilla (Figure 6).

## 3. Results

The results were summarized in the following Table 1, Table 2, Table 3 and Table 4.

## 4. Statistical Analysis

### 4.1. Two-Way Analysis of Variance Model for Anterior Discrepancy

In this statistical study, the relationship between the *y* variable (anterior transverse discrepancy) and sex (M vs. F) in the various observations is analyzed. The estimated model will follow the following expression:

(1) $$y_{ijk}=\;\mu +\alpha_{i}+\gamma_{ij}+\varepsilon_{ij}$$

where:

- *y* is the anterior transverse discrepancy expressed in millimeters.
- μ is the average of the previous transverse discrepancies being analyzed.
- α is the difference parameter of the mean of the ith factor being analyzed.
- γ is the interaction between the factors being analyzed (gender factor and position of the discrepancy).
- ε is the random part of the model on which the following hypotheses are made: (1) that the errors are independent, distributed according to a normal N~(0, $\sigma^{2}$), and (2) that they are homoscedastic.

Hypothesis tests for all parameters will be verified on this model as follows:

(2) $$H_{0}:\;\alpha =0\;H_{1}:\;\alpha \neq 0\;\mathrm{for}\;\mathrm{some}\;i$$

The statistic test to verify the previous system of hypothesis can be obtained by using the so-called analysis of variance 

**Figure 7:**
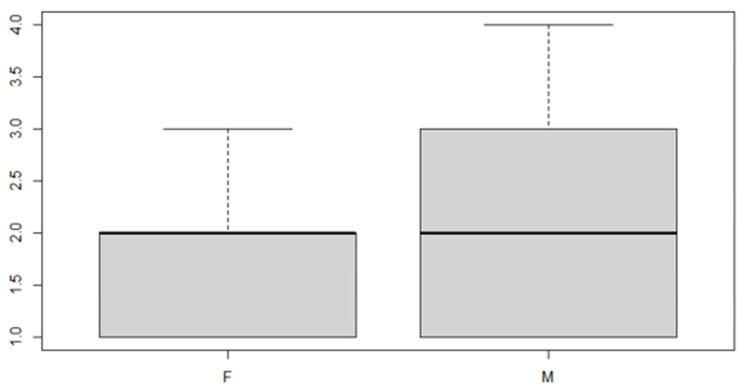
Boxplot for the y variable sex factor.

**Figure 8:**
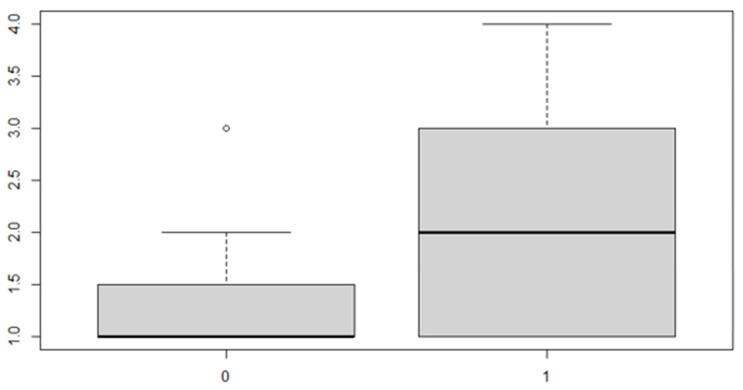
Boxplot for the variable by discrepancy side factor.

In this analysis, we will consider the deviance due to the various factor levels. An ANOVA model is then used in order to achieve the aim of the study. We start with a verification of the mean, the position indices for the sex factor and the location of the discrepancy (Table 5 and Table 6 and Figure 7 and Figure 8).

As can be seen from both the data and the boxplot for sex and the anterior discrepancy side, patients have the same median, but not the same mean. These data would lead to the conclusion that in the ANOVA analysis, the estimated parameter that should highlight the difference between the mean (α) for the factor levels considered could be significant.

A verification of the results obtained from the analysis now follows (Table 7):

It can be noted that the parameter relating to both the "sex" variable and the "side of the anterior discrepancy" variable is significant, but the interaction of the two factors is not significant. Thus, it is necessary to reduce the model, not considering the interaction given that it is null.

To this end, the following data is reported (Table 8 and Table 9):

These results indicate that, in general, the mean of the anterior discrepancy in the sample group is of approximately 1.21 mm. If the patient has an anterior discrepancy on the right side, the value of 0.57 mm should be added to this figure, and if the patient is male, it will be necessary to add an additional 0.30 mm.

**Figure 9:**
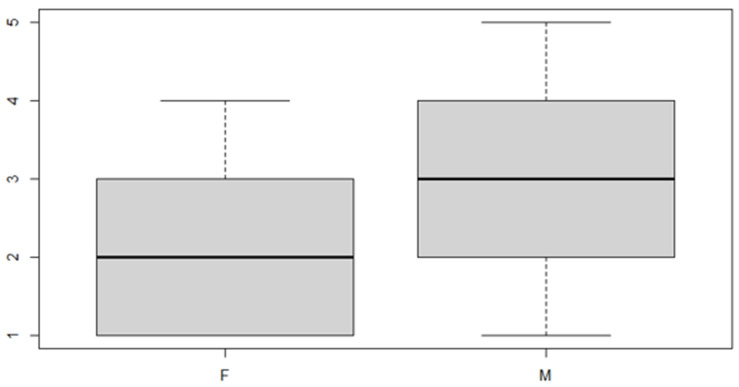
Boxplot for the *y* variable by sex factor.

**Figure 10:**
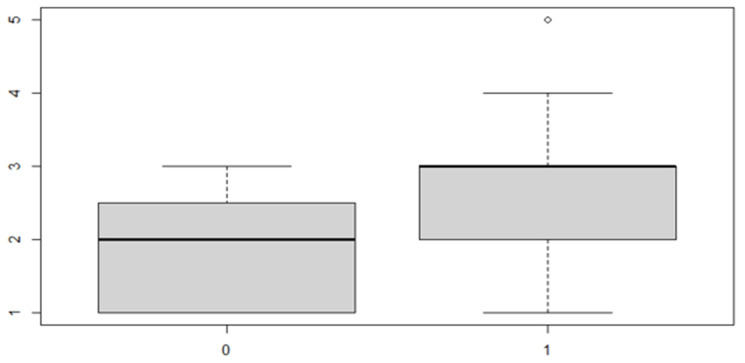
Boxplot for the *y* variable by discrepancy side factor.

The same procedure applies for the posterior discrepancy, whose results are as follows (Table 10 and Table 11 and Figure 9 and Figure 10):

### 4.2. Two-Way Analysis of the Variance Model for Posterior Discrepancy (Results)

In this case, it can be observed, both from the data and from the boxplot for sex and the posterior discrepancy side, that patients have the same median, but not the same mean. This would lead to the conclusion that in the ANOVA analysis, the estimated parameter that should highlight the difference between the mean (α) for the factor levels considered could be significant.

Below can be found the results obtained from the analysis (Table 12):

In this case, the interaction is not significant, so the reduced model is adopted.

The results are reported below (Table 13 and Table 14): 

These results indicate that, in general, the mean of the posterior discrepancy in the sample group is approximately 1.11 mm. If the patient has an anterior discrepancy on the right side, the value of 0.95 mm should be added to this figure, and if the patient is male, an additional 1.5 mm must be added.

### 4.3. Three-Way Analysis of Variance Model for Transverse Discrepancy 

**Figure 11:**
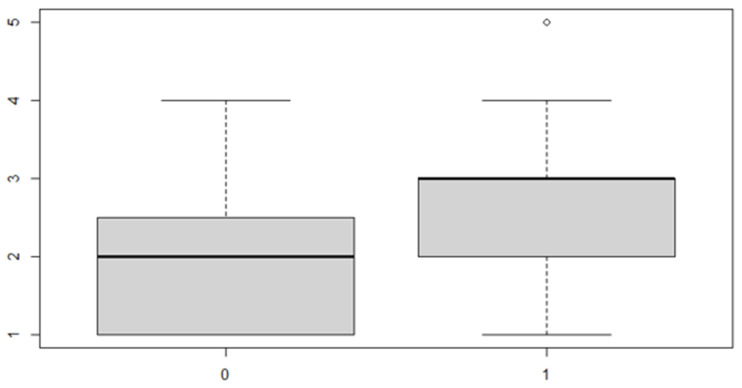
Boxplot for the *y* variable by anterior (0) or posterior (1) discrepancy factor.

**Figure 12:**
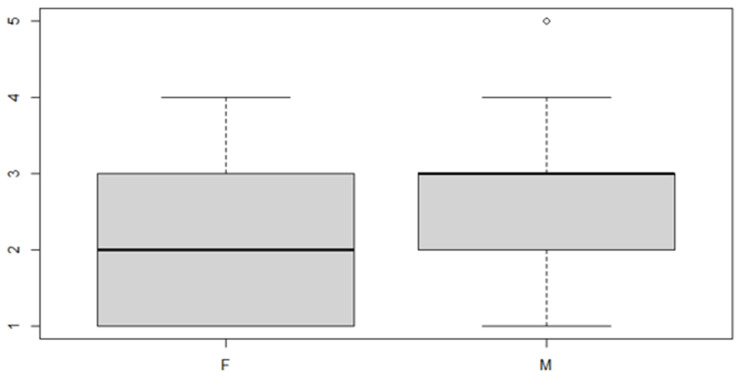
Boxplot for the *y* variable by sex factor.

**Figure 13:**
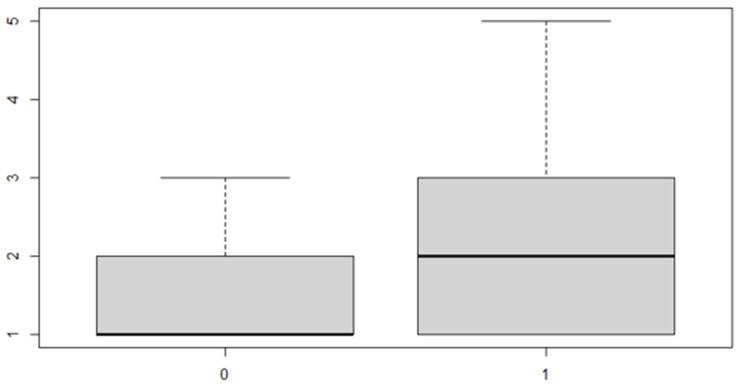
Boxplot for *y* variable by discrepancy side factor.

These results indicate that, in general, the mean of the transverse discrepancy in the sample group is equal to about 0.9373 mm. If the patient has a posterior transverse discrepancy, the value of 0.646 mm should be added, and if it is on the right side, the value of 0.657 mm should be added to the sum of the two numbers indicated above. Finally, if the patient is male, another 0.667 mm must be added (Table 15, Table 16, Table 17 and Table 18 and Figure 11, Figure 12 and Figure 13).

Finally, the information loss test is analyzed if the three-way model is more effective than the models used previously: if the *p*-Value of this test is less than 5%, the three-factor model will be accepted (Table 19):

## 5. Discussion

The developmental type of facial asymmetry is idiopathic and non-syndromic in nature, and it is not uncommonly seen in the general population. The asymmetry is not observed at birth or in infancy, and it appears gradually, usually becoming apparent in the teenage years. On the basis of the results obtained and the relative statistical analysis, it can be deduced that most subjects of this study show transverse asymmetry in one side of the maxillary arch, both in the anterior and posterior part of the arch, with a slight difference between males and females.

The significance of the data shows that males have a greater asymmetry of the maxilla on the horizontal plane than females. The interpretation of the data is as follows. The prevailing chewing side determines a greater expansion of the hemi-maxilla that is subjected to the functional stimuli of the organ, and this expansion concerns males more, given that they express greater muscular strength in the masticatory muscles. This determines a more expanded external rotation of the hemi-maxilla and an internal rotation of the hemi-maxilla of the opposite side. Consequently, changes will take place both at the dental level and at the bone level, involving the entire mandibular–cranial area, especially in growing subjects. Habitual chewing on one side is also responsible for increased skeletal development on the ipsilateral side of bone arches, so the hemi-maxilla that rotates externally will exhibit more mesialized dental elements than the hemi-maxilla that rotates internally, whose dental elements will be more distalized. 

Regarding the bone characteristics, there will be an increase in bone density and thickness at the hemi-maxilla level of the prevailing chewing side, and a greater constriction of the premaxilla on the same side due to a compression action on the interincisal suture. When one side of osseous development is affected, the contralateral side will most inevitably be influenced resulting in a compensational way. So, abnormal muscle function on one side of the masticatory system may cause a constricted maxillary arch or a local factor such as one or more malpositioned teeth.

Regarding the mandible, this will have a more voluminous condyle on the working side and it will become hypertrophic. 

Examining the adjustment on the skull, the external rotation of the hemi-maxilla will occur on the working side, and this rotation will affect the kinetics of the occipitotemporal region. In particular, by carrying out the anterior occipital rotation, there will also be an anterior rotation of the temporal squama and a verticalization of this will also occur. This torque effect will increase the external rotation of the jaw, and vice versa. The sum of the effects of growth and function will determine craniofacial harmony or give rise to varying degrees of asymmetry on the hard and soft tissues. 

In this regard, it is appropriate to differentiate between cranial asymmetries, which involve the skull in all or most of its components, from maxillo-mandibular asymmetries, which are localized selectively on the maxillary bones. Skull asymmetries are mainly associated to congenital or hereditary malformation syndromes resulting from alterations occurring during intrauterine development or to a decreased genetic control during the formation of the bilateral structures of the skull, making the subject more vulnerable to the actions of environmental factors.

The maxillo-mandibular asymmetries, on the other hand, are localized at the level of the maxillaries and therefore at the level of the lower third of the face. Such asymmetries can be skeletal, functional or dental in nature.

The congenital etiology of mandibular skeletal asymmetries includes alterations in the normal developmental process of the maxillary bones occurring during the period of intrauterine life that do not necessarily result from a gene mutation. Facial deformations can be caused by abnormal pressure on the jaws due to the position of the fetus in the intrauterine space, or they could occur during delivery. The postnatal development of any craniofacial region is linked to the interactions of structural counterparts. In this way, the part and counterpart expand to the same extent and, consequently, growth is mutually balanced and therefore harmonious. With regard to the acquired causes, in most cases, they can be attributed to an anomalous and unbalanced distribution of forces on the maxillary bones, as a result of which the counterparts are unbalanced, especially if these events occur in the developmental age. In this case, the asymmetry will tend to affect the skeleton, and therefore it will establish itself permanently, becoming irreversible, unless a surgical correction is carried out. So, an important condition that leads to asymmetry in the upper hemi-maxilla is unilateral chewing—that is, a functional attitude with a neuromuscular prevalence of one half compared to the contralateral one, which remains passive. Some authors such as Kim [22] and Gribel [23] claim about asymmetry that lateral guidance is most predominant on the left side of the face. This occurrence could be explained by the dominant growth potential on the right side of the face, particularly considering the larger dimensions of the skull and the brain of individuals on the right side. Galland [24] validated the functional-masticatory hypothesis and demonstrate that the biomechanical changes associated with dietary change strongly affect the jaws and the aspects of facial morphology. Van Spronsen [25] stated that the masticatory performance of subjects with a long-face craniofacial morphology is considerably reduced compared with subjects with a vertically normal skull form. He confirmed that tensile loadings evoked by contracting jaw muscle fibers attached to the periosteum, may stimulate local bone apposition. So, it can be hypothesized that an increased function of the masseter and temporalis muscles, being attached laterally to the ramus, the zygomatic arches, and the temporal bones, will stimulate bone apposition in these areas, consequently leading to an increase in craniofacial width. Furthermore, masticatory muscle loading will be transferred through the dentition and the temporomandibular joints to the craniofacial complex and indirectly influence its growth and strength. So, he concluded that the dimensions and morphology of the facial bones as well as the sutural bone apposition were negatively affected by reduced masticatory function. 

The reduction of the hemiarch diameter in the maxilla derives from a reduction in masticatory load on that side. As confirmed by other authors (Sella-Tunis [26], Eyquem [27] and Toro-Ibacache [28]), a human mandibular bone shape is associated with masticatory muscle force. A good example are those subjects suffering with bruxism showing broad jaws, whereas those with weak bite force or no force in one hemimaxilla arch have narrow jaws or a reduction of the hemiarch diameter in the maxilla. So, when only one side of the jaw is used in mastication, no function stimuli are applied and a reduction of the alveolar bone happens. The reduction of masticatory load intensity resulting from dietary habits, or resulting from unilateral mastication habits has been proposed by various authors as important factors that affect the craniofacial morphology [29], [30]. Softer and more processed foods are widely hypothesized to lead to less facial growth, especially in the lower face and the alveolar crests, because of the potential effects of force-generated strain. Strain can stimulate periosteal growth, while low magnitudes and frequencies of loading can lead to local bone resorption. Herring [31] has shown that young adults with larger muscle cross-sectional areas and/or higher bite forces have larger, less variably-sized faces than those who produce less bite force. During unilateral mastication, the zygomatic arch and postorbital septum are subject to bending (Ross, [32]) while the mandible is subject to a combination lateral transverse and sagittal bending, and twisting about the longitudinal axis (Varrela, [33]). It seems that in the zygomatic arch, strain generated by hard food is approximately twice that of cooked food, so some authors supported this concept by studies demonstrating that masticating softer, more processed (cooked) foods as in humans can lead to reduced facial growth. For example, Holmes [34] studied a role for functional mechanical loading of the mandible during growth in producing adult differences in mandibular corpus morphology. Agrawal [35] found a clear relationship between the electrical activity of a jaw-closing muscle and the mechanical properties of food for the first time. Furthermore, Susan Herring [36] applied these concepts not only to humans, but to all mammals. She said that an asymmetrical muscle usage during mastication sets up torques on the skull bones and combines with occlusal loads to produce bony deformations not only in the tooth-bearing jaw bones, but also in more distant elements. 

Finally, the most important point of an orthodontic approach is the diagnosis. So, if the clinician does not habitually evaluate the maxilla symmetry on the horizontal plane, many asymmetries will be misunderstood and, therefore, untreated. By a treatment point of view, the majority of appliances, especially fixed orthodontic appliances, work in a symmetrical way. It means that if there is an asymmetry, perhaps in the maxilla, the orthodontic appliance does not correct that asymmetry, unless the appliance is working in an asymmetrical way. So, in growing subjects a lot of skull asymmetries are functional, for example, due to a unilateral mastication. Those subjects show various signs of functional asymmetries, perhaps at orbital level or on the ears lips, smile, and so on. Therefore, as the orthodontist must find a maxillary asymmetry on the horizontal plane in growing patients, they must use an asymmetrical appliance in order to get harmony and equilibrium of form and function. 

## 6. Conclusions

Very few studies about an asymmetry in the horizontal plane on the upper maxillary jaw were carried out. A unilateral mastication causes an asymmetric shape in the maxilla consisting of more expansion, and larger hemiarch diameter in that side that receives functional stimuli. Since a very few growing subjects have a bilateral mastication, so, many asymmetries are in the maxillary bone, but they are misunderstood. In this work, the authors found a lot of asymmetries in the maxilla. They analyzed the hemiarch maxilla measurement in males and females. Most subjects show transverse asymmetry in both the anterior and posterior arches, indistinctly, in both males and females. The most frequent occurrence that leads to asymmetry in the upper left hemi-maxilla with respect to the contralateral is unilateral chewing—that is, a functional attitude with a neuromuscular prevalence of one half compared to the contralateral one, which remains passive. This study suggests to always consider the measurement of the hemiarches in the maxilla, especially in growing patients, so proper appliances by the orthodontists will be used in order to reach a balance of chewing forces, symmetry of craniofacial bones, and a good harmony of soft tissues. Finally, asymmetry treatment depends on the patient’s age, etiopathogenesis, and on the degree of disharmony, and might include from asymmetrical orthodontic mechanics to orthognathic surgery. Thus, the present study aims at addressing important aspects to be considered by the orthodontist reaching an accurate diagnosis and treatment plan of facial asymmetry, in addition to reporting treatment of some patients’ carriers of such a challenging disharmony.

## Figures and Tables

**Table 1:** Anterior transverse asymmetry in females.

pts.	Mean of Right Anterior Hemiarch Diameter and SD	Mean of Left Anterior Hemiarch Diameter and SD	Location of Discrepancy
72	17.46 mm SD = 2.88 mm	18.86 mm SD = 3.37 mm	R/L

**Table 2:** Posterior transverse asymmetry in females.

pts.	Mean of Right Posterior Hemiarch Diameter and SD	Mean of l Left Posterior Hemiarch Diameter and SD	Location of Discrepancy
72	21.85 mm SD = 2.76	22.58 mm SD = 3.14	R/L

**Table 3:** Anterior transverse asymmetry in males.

pts.	Means of Right Anterior Hemiarch Diameter and SD	Mean of Left Anterior Hemiarch Diameter and SD	Location of Discrepancy
72	17.85 mm SD = 2.63	18.92 mm SD = 3.38	L/R

**Table 4:** Posterior transverse asymmetry in males.

pts.	Mean of Right Posterior Hemiarch Diameter and SD	Mean of l Left Posterior Hemiarch Diameter and SD	Location of Discrepancy
72	22.38 mm SD = 2.83	23.42 mm SD = 3.27	R/L

**Table 5:** Sex variable.

**F**					
**Min**	**1st Qu**	**Median**	**Mean**	**3rd Qu.**	**Max**
1.000	1.000	2.000	1.716	2.000	3.000
**M**					
**Min**	**1st Qu**	**Median**	**Mean**	**3rd Qu.**	**Max**
1.000	1.000	2.000	2.041	3.000	4.000

**Table 6:** Discrepancy side variable.

**Left Side**					
**Min**	**1st Qu**	**Median**	**Mean**	**3rd Qu.**	**Max**
1.000	1.000	1.000	1.333	1.250	3.000
**Right Side**					
**Min**	**1st Qu**	**Median**	**Mean**	**3rd Qu.**	**Max**
1.000	1.000	2.000	1.938	3.000	4.000

**Table 7:** Analysis of variance table.

Response: y				
	Df Sum	Sq Mean	Sq F Value	Pr (>F)
side	14.005	4.0048	6.1284	0.01453 *
sex	13.219	3.2194	4.9265	0.02810 *
side:sex	10.074	0.0745	0.114	0.73619
Residuals		136	88.873	0.6535

Signif. codes: 0.01 ‘*’.

**Table 8:** Residuals.

Min	1Q	Median	3Q	Max
−1.08009	−0.77590	−0.08009	0.82504	1.91991

**Table 9:** Coefficients.

	Estimate	Std.	Error *t* Value	Pr(>|t|)
(Intercept)	1.2066	0.2395	5.039	1.4 × 10^−6^ ***
right	0.5693	0.2438	2.335	0.0210 *
sex M	0.3042	0.1366	2.227	0.0276 *

Signif. codes: 0 ‘***’ 0.01 ‘*’. Residual standard error: 0.8058 on 137 degrees of freedom. Multiple R-squared: 0.07512—Adjusted R-squared: 0.06162—F-statistic: 5.563 on 2 and 137 DF—*p*-value: 0.004753.

**Table 10:** Sex variable.

**F**					
**Min.**	**1st Qu.**	**Median**	**Mean**	**3rd Qu.**	**Max.**
1.00	1.00	2.00	2.03	3.00	4.00
**M**					
**Min.**	**1st Qu**	**Median**	**Mean**	**3rd Qu.**	**Max.**
1.000	2.000	3.000	3.043	4.000	5.000

**Table 11:** Discrepancy side variable.

**Left Side**					
**Min**	**1st Qu**	**Median**	**Mean**	**3rd Qu.**	**Max**
1.000	1.000	2.000	1.857	2.500	3.000
**Right Side**					
**Min**	**1st Qu**	**Median**	**Mean**	**3rd Qu.**	**Max**
1.000	2.000	3.000	2.585	3.000	5.000

**Table 12:** Analysis of variance table.

Response: y				
	Df Sum	Sq Mean	Sq F value	Pr (>F)
side	13.515	3.515	4.0028	0.04746 *
sex	137.594	37.594	42.8082	1.19 × 10^−9^ ***
side:sex	10.032	0.032	0.0369	0.84800
Residuals		133	116.800	0.878

Signif. codes: 0 ‘***’ 0.01 ‘*’. 0.05 ‘.’ 0.1 ‘ ’ 1.

**Table 13:** Residuals.

Min	1Q	Median	3Q	Max
−2.1109	1.0583	0.1109	0.8891	1.9417

**Table 14:** Coefficients.

	Estimate	Std.	Error *t* Value	Pr (>|t|)
(Intercept)	1.1053	0.3710	2.979	0.00344 **
right	0.9530	0.3639	2.619	0.00984 **
sex M	1.0526	0.1603	6.566	1.0 × 10^−9^ ***

Signif. codes: 0 ‘***’ 0.001 ‘**’ 0.05 ‘.’ 0.1 ‘ ’ 1. Residual standard error: 0.9337 on 134 degrees of freedom. Multiple R-squared: 0.2603—Adjusted R-squared: 0.2492. F-statistic: 23.57 on 2 and 134 DF—*p*-value: 1.688 × 10^–9^.

**Table 15:** Anterior or posterior factor.

**Anterior**					
**Min.**	**1st Qu.**	**Median**	**Mean**	**3rd Qu.**	**Max.**
1.000	1.000	2.000	1.886	2.250	4.000
**Posterior**					
**Min.**	**1st Qu.**	**Median**	**Mean**	**3rd Qu.**	**Max.**
1.000	2.000	3.000	2.547	3.000	5.000

**Table 16:** Sex factor.

**F**					
**Min.**	**1st Qu.**	**Median**	**Mean**	**3rd Qu.**	**Max.**
1.000	1.000	2.000	1.873	2.750	4.000
**M**					
**Min.**	**1st Qu.**	**Median**	**Mean**	**3rd Qu.**	**Max.**
1.000	2.000	3.000	2.531	3.000	5.000

**Table 17:** Side factor.

**Left Side = 0**						
**Min.**		**1st Qu.**	**Median**	**Mean**	**3rd Qu.**	**Max.**
1.000		1.000	1.000	1.526	2.000	3.000
**Right Side = 1**						
**Min.**	**1st Qu.**		**Median**	**Mean**	**3rd Qu.**	**Max.**
1.000	1.000		2.000	2.264	3.000	5.000

**Table 18:** Analysis results.

Residuals:				
Min	1Q	Median	3Q	Max
−1.9072	−0.5943	−0.2402	0.7388	2.0928
**Coefficients:**				
	**Estimate**	**Std.**	**Error t value**	**Pr(>|t|)**
(Intercept)	0.9373	0.2154	4.352	1.91 × 10^−5^ ***
side1	0.6570	0.2119	3.100	0.00213 **
sex M	0.6669	0.1069	6.237	1.69 × 10^−9^ ***
post. 1	0.6460	0.1071	6.030	5.31 × 10^−9^ ***
---				

Signif. codes: 0 ‘***’ 0.001 ‘**’. Residual standard error: 0.8893 on 273 degrees of freedom. Multiple R-squared: 0.241—Adjusted R-squared: 0.2326. F-statistic: 28.89 on 3 and 273 DF—*p*-value: 2.963 × 10^–16^.

**Table 19:** Analysis of variance table.

Model 1: y~Side + Sex + Bin				
Model 2: y~Side + Sex				
Res.Df	RSS Df	Sum of Sq	F	Pr (>F)
1	273	215.89		
2	274	244.65 × 10^−1^	−28.755	36.362 5.306 × 10^−9^ ***

Signif. codes: 0 ‘***’. The *p*-value is less than 5%, therefore the three-factor model is accepted.

## Data Availability

The datasets analyzed during the current study are available from the corresponding author on request.
